# Preventive Dental Visits Among Older Adults With Mental Health Conditions: A Retrospective Cohort Study From a Dental School Setting

**DOI:** 10.1111/scd.70122

**Published:** 2025-12-04

**Authors:** Katie McAllister, Fang Qian, Jodi Tate, Leonardo Marchini

**Affiliations:** ^1^ The University of Iowa College of Dentistry and Dental Clinics Iowa Iowa USA; ^2^ Division of Biostatistics and Computational Biology The University of Iowa College of Dentistry and Dental Clinics Iowa Iowa USA; ^3^ Department of Psychiatry The University of Iowa Carver College of Medicine Iowa Iowa USA; ^4^ Department of Preventive and Community Dentistry The University of Iowa College of Dentistry and Dental Clinics Iowa Iowa USA

**Keywords:** dental health services, health records, mental disorders, older adults, preventive dentistry

## Abstract

**Objective:**

To assess the frequency of annual preventive dental visits among older adults with self‐reported mental health conditions receiving care at a dental school and to identify the variables associated with visit prevalence.

**Methods:**

Data were extracted from electronic health records for all individuals aged 65 years or older who self‐reported a mental health condition between 2008 and 2024. Demographic and clinical characteristics of the participants were summarized using descriptive statistics, and significant predictors of annual preventive dental visits were identified through a multivariable logistic regression analysis.

**Results:**

A total of 3846 subjects met the inclusion criteria. From this total, 1,853 (48.2%) received preventive dental services and 1,294 (33.6%) had at least one annual preventive dental visit. Within this group, 64.2% were female, the average age was 76.0 ± 9.1 years, 54% were self‐paying, 62% resided within 30 miles of the clinic, and 70.9% were treated by pre‐doctoral students. Nearly 43% were obese, and they reported taking an average of 7.8 ± 7.2 daily medications. The average number of teeth was 25.0 ± 7.3. Additionally, 83.7% had heart disease, 54.9% experienced xerostomia, and 5.3% reported a history of illicit drug addiction. Approximately 40% used tobacco, 45.7% had diabetes, and 30.7% struggled with alcohol addiction. Each additional year of age was associated with a 4% increase in the odds of attending two or more annual preventive dental visits (OR = 1.04, 95% CI: 1.01–1.08; *p* = 0.004). Additionally, patients treated by faculty members had significantly higher odds of attending two or more annual preventive dental visits compared to those treated by predoctoral students (OR = 3.77, 95% CI: 2.24–6.36; *p* < 0.001).

**Conclusion:**

In this sample of older adults with self‐reported mental health condition, about one‐third had at least one annual preventive dental visit during the observation period. Receiving care from faculty members was significantly associated with increased odds of attending annual preventive dental visits.

## 1 Introduction

As the global population continues to age, the need for accessible and age‐appropriate oral health care among older adults is becoming increasingly critical [1]. However, individuals aged 65 years and older face unique challenges in maintaining oral health, many of which are compounded by chronic systemic conditions, medication use, physical and cognitive impairments, and mental health disorders [2]. Mental health conditions are common among older adults in the United States. An estimated 11.4% of this population experience an anxiety disorder each year, 6.8% are affected by a mood disorder, 3.8% meet the criteria for a substance use disorder, and 14.5% are diagnosed with at least one personality disorder [3]. Despite their prevalence, these conditions are frequently underdiagnosed and untreated [4], yet they have significant implications for health‐related behaviors and outcomes, including the maintenance of oral health [5].

A growing body of literature supports a bidirectional relationship between mental health and oral health [6, 7]. Mental disorders and oral diseases are influenced by shared social and intermediate determinants and arise from complex interactions among social, psychological, behavioral, and biological factors. These conditions also influence one another through multiple bidirectional pathways involving the same interconnected processes [7].

Mental disorders are often linked to behaviors that negatively impact oral health, including tobacco and alcohol use, poor dietary habits, and inadequate oral hygiene [5, 8]. Individuals may turn to substances like nicotine and alcohol as a form of self‐medication to cope with psychological distress [9]. Additionally, conditions such as depression can contribute to disordered eating patterns, increased sugar consumption, and overall poor nutritional choices [10]—all of which can further compromise oral health.

Conversely, oral diseases may contribute to the development or worsening of mental health conditions through several interconnected mechanisms. These include neuroinflammatory responses, chronic psychological stress resulting from diminished quality of life, social stigma, and discrimination, as well as the impact of oral health problems on nutritional status [7].

Oral diseases may contribute to neuroinflammation through several mechanisms: by triggering chronic systemic inflammation, by allowing oral bacteria or their toxins to enter the brain through a compromised blood‐brain barrier, or by enabling communication between oral bacteria and brain‐resident immune cells [11]. Inflammatory mediators released from oral infection sites can circulate in the bloodstream, potentially leading to systemic inflammation that has been linked to mental health conditions such as depression [12] and schizophrenia [13]. Oral diseases can also negatively impact self‐esteem, social interaction, and quality of life, potentially contributing to mental health issues [14]. Stigma related to poor dental appearance may lead to discrimination and reduced access to care, further exacerbating psychological distress [15]. In addition, oral health issues, especially tooth loss, can impair eating ability and lead to poor intake of essential nutrients like fiber, protein, vitamins, and minerals—factors that are known to influence the onset and progression of various mental health conditions [16].

Despite these associations, limited research has examined how mental health may influence patterns of dental care utilization in older adults, particularly regarding their adherence to preventive dental appointments [17]. Regular preventive dental visits are essential for early detection of oral diseases and for providing tailored preventive interventions, yet attendance can be inconsistent in this population [18]. Preliminary research associated with “poor mental health days” and frequency of dental appointment adherence shows that these individuals are more likely to delay dental appointments, which might lead to worse dental outcomes [19].

A deeper understanding of how mental health affects older adults—and its influence on their oral health—is essential for improving dental care for this underserved and rapidly growing population in the United States. This study aims to examine the frequency of annual preventive dental visits among dental patients aged 65 and older who have self‐reported mental health conditions and to identify the factors associated with their likelihood of returning for routine preventive care. We hypothesized that older adults with self‐reported mental health conditions would have fewer annual preventive dental visits than younger older adults with similar conditions. We also anticipated that factors such as age, distance to the clinic, provider type, and the presence of comorbidities would be significantly associated with the frequency of annual preventive dental visits.

## 2 Materials and Methods

The University of Iowa Institutional Review Board determined that this project did not meet the regulatory definition of human subjects’ research and therefore did not require full IRB review (Protocol #202405403). Following this determination, a query was conducted in the University of Iowa College of Dentistry and Dental Clinics’ electronic health records (EHR) to identify patients who met the following inclusion criteria: (1) aged 65 years or older at the time of their first recorded dental visit during the study period (2008–2024), (2) had at least one self‐reported mental health condition, and (3) received at least one preventive dental visit per year.

Mental health condition was defined solely based on patient self‐report in the EHR via the intake question: “Do you have, or have you had mental health issues?” with a response of “Yes.” No ICD codes or provider‐entered diagnoses were used for classification. While self‐report may introduce some misclassification, this approach reflects the patient‐reported status documented in our EHR. To ensure plausibility, responses were checked for consistency across repeated visits, and available clinical notes were reviewed when possible; nonetheless, some measurement error may remain.

De‐identified data were extracted from the EHR and provided to the research team in an Excel file by a member of the information technology team. Patient demographic and health history variables extracted from the EHR included age (analyzed as a continuous variable or categorized as 65–74 and ≥75 years) and gender (female or male). Health history variables included body mass index (BMI; <30 = non‐obese, ≥30 = obese), number of daily medications (analyzed as a continuous variable and categorized as 0–3, 4–10, or ≥11), number of teeth present, and presence or absence of tobacco use, alcohol or drug addiction, diabetes, dry mouth, and heart disease. Additional variables included distance from the patient's residence to the college (analyzed as a continuous variable and categorized as <30, 31–60, or >60 miles), type of dental insurance (self‐pay or non–self‐pay), presence or absence of a medical power of attorney, and type of dental provider (predoctoral student, resident, or faculty member). These variables were selected from the available EHR data to describe the patient population and identify factors potentially associated with the frequency of preventive dental visits.

The frequency of annual preventive dental visits was evaluated for the observation period (2008–2024). To be included in the analysis, patients were required to have at least one preventive dental treatment during each year of observation. The primary outcome, the mean annual frequency of preventive dental visits, was dichotomized as fewer than two visits (<2) versus two or more visits (≥2) per year. The cutoff of two visits per year was selected because semiannual preventive dental visits represent the widely accepted standard of care for maintaining oral health and are commonly used in prior studies evaluating dental care utilization.

Descriptive statistics were used to summarize patient demographic and clinical characteristics. Categorical variables were presented as frequencies and percentages, while continuous variables were summarized using means and standard deviations (SD) or medians and ranges, as appropriate. Simple logistic regression analyses were performed to examine associations between the frequency of annual preventive dental visits (<2 visits vs. ≥2 visits per year) versus each of demographic or clinical variable. Unadjusted *p* values, odds ratios (ORs), and 95% confidence intervals (CIs) were reported.

Multivariable logistic regression analyses were conducted to identify factors independently associated with having two or more annual preventive dental visits. A theory‐driven model‐building approach included patient demographics, insurance type, geographic access, provider type, medical power of attorney, health history, and calendar period (pre‐COVID, COVID‐19 disruption, and post‐COVID recovery).

Three nested logistic regression models were fitted: (1) a full model including all theory‐driven covariates (Model 1), (2) a reduced model excluding variables with >30% missing data (BMI and dry mouth (Model 2), and (3) a model including only the calendar period and variables that were statistically significant (*p* < 0.05) in simple logistic regression analyses (Model 3).

Model performance was evaluated using the –2 log likelihood, Akaike Information Criterion (AIC), and Hosmer–Lemeshow goodness‐of‐fit test for calibration. Discriminative ability was assessed using the area under the receiver operating characteristic curve (AUC), with 95% CIs computed via DeLong's method. Pairwise comparisons of AUCs between models were also conducted using DeLong's test to assess statistical differences in discrimination.

In addition, multicollinearity among predictors was checked using variance inflation factors (VIFs) and tolerance values (TOL), and selected possible two‐way interactions (e.g., age × provider, distance × provider, age × number of daily medications) were considered in the final model.

All statistical tests were two‐sided, with a significance level of *α* = 0.05. Analyses were conducted using the SAS System, version 9.4 (SAS Institute Inc., Cary, NC, USA).

## 3 Results

**FIGURE 1:**
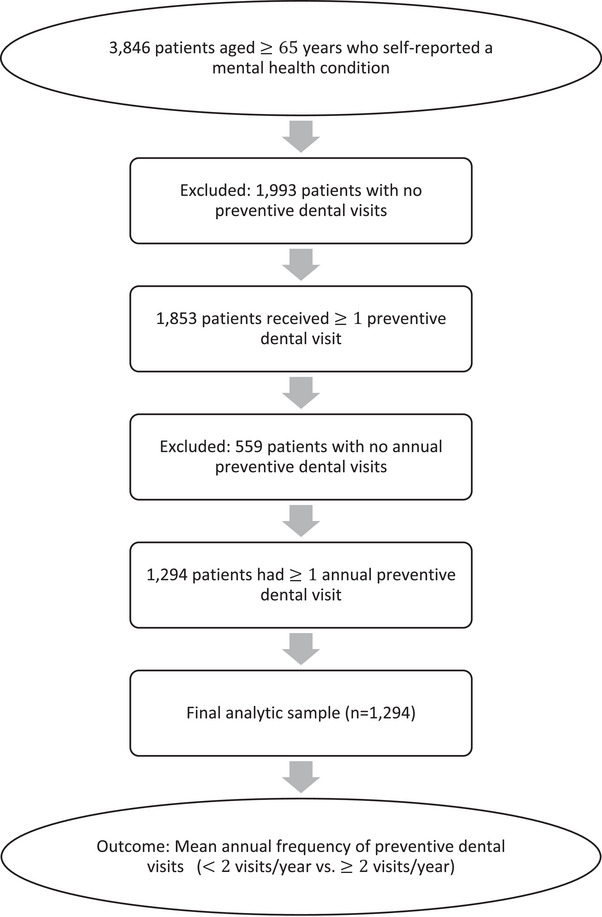
Flow diagram of participant selection.

A total of 3,846 patients aged 65 or older who self‐reported a mental health condition were identified in the EHR between 2008 and 2024. Of these, 1,853 (48.2%) received at least one preventive dental treatment, and 1,294 (33.6% of the original sample) had at least one annual preventive dental visit, comprising the analytic sample. Patients without annual preventive visits (*n* = 559) were excluded, as the analysis focused on factors associated with the frequency of annual preventive dental visits. Figure 1, titled “Flow diagram of participant selection,” illustrates this process.

The outcome variable was the mean annual frequency of preventive dental visits, categorized as fewer than two visits per year versus two or more visits per year during the study period. Notably, only 542 patients, representing 14.1% of the original sample, had two or more preventive appointments every year.

Of the 1,294 patients included in the study, 64.2% were female, with an age of 76.0 ± 9.1 years; 46% were 75 or older. Approximately 54% were self‐paying, and the majority (70.9%) were treated by pre‐doctoral students. About 62% resided within 30 miles of the dental school. Bivariate analyses showed that patients with ≥2 annual preventive dental visits were significantly younger (mean age 74.3 ± 7.9 vs. 77.3 ± 9.7 years, *p* < 0.001), more likely to be aged 65–74 (*p* < 0.001), and more likely to live within 30 miles of the clinic (*p* = 0.013). Provider type was also associated with visit frequency: those with ≥2 visits were more often treated by faculty, while those with <2 visits were more often seen by students (*p* < 0.001). No significant differences were observed by gender, insurance type, or medical power of attorney status (Table 1).

**TABLE 1:** Demographic characteristics of participants and their associations with the frequency of annual preventive dental visits.

		Annual preventive dental visits			
Demographics	All participants (*N* = 1,294)	<2 Visits (*N* = 752) *n* (%)	≥2 Visits (*N* = 542) *n* (%)	Unadjusted OR (CI)	*p* value
Gender					0.974
Female	830 (64.2)	483 (58.2)	347 (41.8)	1.00 (0.79–1.25)	
Male	463 (35.8)	269 (58.1)	194 (41.9)	Ref.	
Age at last treatment (years)					<0.001^*^
Mean ± SD	76.0 ± 9.1	77.3 ± 9.7	74.3 ± 7.9	0.96 (0.95–0.98)	
Median (range)	74 (65–104)	75 (65–103)	72 (65–104)		
Age group					<0.001^*^
65–74 years	699 (54.0)	365 (52.2)	334 (47.8)	1.70 (1.36‐2.13)	
75+ years	595 (46.0)	387 (65.0)	208 (35.0)	Ref.	
Types of insurance					0.141
Self‐pay	702 (54.3)	421 (60.0)	281 (40.0)	0.85 (0.68–1.06)	
Non‐self‐pay (AG+DWP+INS+XIX)	592 (45.7)	331 (55.9)	261 (44.1)	Ref.	
Presence of a medical a power of attorney					0.613
Yes	42 (3.3)	26 (61.9)	16 (38.1)	0.85 (0.45–1.60)	
No	1252 (97.7)	726 (58.0)	526 (42.0)	Ref.	
Distance from patient's residence to the UICOD (miles)					0.508
Mean ± SD	38.2 ± 61.5	37.3 ± 64.8	39.6 ± 56.6	1.00 (0.98–1.01)	
Median (range)	24.5 (1–1332)	21.0 (1–1332)	26.0 (1–708)		
Distance from patient's residence to the dental school					0.038^*^
60+ miles	263 (20.3)	139 (52.8)	124 (47.2)	1.38 (1.04–1.85)	(0.028^*^)
31–60 miles	227 (17.6)	124 (54.6)	103 (45.4)	1.29 (0.95–1.75)	(0.110)
Less than or equal to 30 miles	804 (62.1)	489 (60.8)	315 (39.2)	Ref.	
Types of providers					<0.001^*^
Faculty member	185 (18.8)	58 (31.4)	127 (68.6)	2.89 (2.02–4.15)	(<0.001^*^)
Resident	102 (10.3)	57 (55.9)	45 (44.1)	1.04 (0.67–1.61)	(0.935)
Pre‐doctoral student	700 (70.9)	398 (56.9)	302 (43.1)	Ref.	
Calendar‐period effects					0.489
Pre‐Covid baseline period (2008–2019)	569 (44.0)	341 (59.9)	228 (40.1)	0.87 (0.68–1.10)	(0.288)
Covid‐19 disruption period (2020–2021)	218 (16.8)	125 (57.3)	93 (42.7)	0.96 (0.70–1.33)	(.0.819)
Post‐Covid recovery period (2022–2024)	507 (36.2)	286 (56.4)	221 (43.6)	Ref.	

*Notes*: Results are from separate simple logistic regression models evaluating factors associated with having ≥2 annual preventive dental visits. Each row represents an independent model. “Ref” indicates the reference category. *p* < 0.05 was considered statistically significant (*).

Abbreviations: CI, 95% Wald confidence interval; OR, odds ratio.

Among these patients, nearly 43% were classified as obese, with an average of 7.8 ± 7.2 daily medications and 25.0 ± 7.3 teeth present. Additionally, 83.7% of the patients had heart disease, 54.9% experienced dry mouth, and 5.3% had a history of drug addiction. Approximately 40% of the patients used tobacco, 45.7% had diabetes, and 30.7% struggled with alcohol addiction. Compared to those with <2 visits, patients with ≥2 visits were significantly more likely to report tobacco use (*p* = 0.003) and alcohol addiction (*p* < 0.001), and less likely to have heart disease (*p* = 0.003) and diabetes (*p* = 0.028). No significant differences were found for number of medications, BMI, number of teeth, drug addiction, or dry mouth (*p* > 0.05) (Table 2).

**TABLE 2:** Clinical characteristics of participants and their associations with the frequency of annual preventive dental visits.

		Annual preventive dental visits			
Clinical characteristics	All participants (*N* = 1,294)	<2 Visits (*N* = 752) *n* (%)	≥2 Visits (*N* = 542) *n* (%)	Unadjusted OR (CI)	*p* value
Number of daily medications					0.641
Mean year ±SD	7.8 ± 7.2	7.9 ± 7.2	7.7 ± 7.2	0.99 (0.98–1.01)	
Median (Range)	7 (0–54)	7 (0–54)	6 (0–43)		
Number of daily medications					0.872
0–3	417 (32.2)	238 (57.1)	179 (42.9)	1.06 (0.80–1.42)	(0.602)
4–10	517 (40.0)	303 (58.6)	214 (41.4)	1.00 (0.76–1.31)	(0.785)
11+	360 (27.8)	211 (58.6)	149 (41.4)	Ref.	
BMI Level (kg/m^2^)					0.403
<30 (Non‐obesity)	504 (57.2)	259 (51.4)	245 (48.6)	0.89 (0.68–1.17)	
≥30 (Obesity)	377 (42.8)	183 (48.5)	194 (51.5)	Ref.	
Number of teeth present					0.121
Mean ± SD	25.0 ± 7.3	24.7 ± 7.5	25.5 ± 7.1	1.02 (0.99–1.03)	
Median (Range)	27 (0–32)	26 (0–32)	27 (0–32)		
Tobacco use					0.003^*^
Yes	430 (40.1)	206 (47.9)	224 (52.1)	1.46 (1.14–1.86)	
No	643 (59.9)	368 (57.2)	275 (42.8)	Ref.	
Presence of diabetes					0.028^*^
No	653 (54.3)	349 (53.5)	304 (46.5)	1.29 (1.03–1.63)	
Yes	549 (45.7)	328 (59.7)	221 (40.3)	Ref.	
Alcohol addiction					<0.001^*^
Yes	328 (30.7)	148 (45.1)	180 (54.9)	1.63 (1.26–2.12)	
No	740 (69.3)	424 (57.3)	316 (42.7)	Ref.	
Drug addiction					0.494
Yes	57 (5.3)	28 (49.1)	29 (50.9)	1.21 (0.71–2.05)	
No	1012 (94.7)	544 (53.7)	468 (46.3)	Ref.	
Presence of heart disease					0.003^*^
No	207 (16.3)	100 (48.3)	107 (51.7)	1.58 (1.17–2.13)	
Yes	1067 (83.7)	636 (59.6)	431 (40.4)	Ref.	
Presence of dry mouth					0.574
Yes	391(53.9)	192 (49.1)	199 (50.9)	1.09 (0.81–1.46)	
No	334 (46.1)	171 (51.2)	163 (48.8)	Ref.	

*Notes*: Results are from separate simple logistic regression models evaluating factors associated with having ≥2 annual preventive dental visits. Each row represents an independent model. “Ref” indicates the reference category.

* *p* < 0.05 was considered statistically significant.

Abbreviations: CI, 95% Wald confidence interval; OR, odds ratio.

Logistic regression Model 1 demonstrated the best overall model fit in the complete‐case subsample, with the lowest –2 log‐likelihood (888.7) and AIC (930.7) (Table 3). Models 2 and 3, based on larger analytic samples, had higher –2 log‐likelihoods (1222.4 and 1230.7) and comparable AICs (1260.4 and 1254.7, respectively). Calibration across all models was acceptable, with non‐significant Hosmer–Lemeshow tests (*p* = 0.42–0.69), suggesting no evidence of model misfit.

**TABLE 3:** Multivariable logistic regression analyses for the factors associated with frequency of annual preventive dental visits.

	Model 1^a^	Model 2^b^	Model 3^c^
Variable	OR (95% CI)	OR (95% CI)	OR (95% CI)
Gender	NS	NS	Not included
Female			
Male			
Age at last treatment (years)	1.05 (1.01–1.08)**	NS	NS
Types of insurance	NS	NS	Not included
Self‐pay			
Non‐self‐pay (AG+DWP+INS+XIX)			
Presence of a medical a power of attorney	NS	NS	Not included
Yes			
No			
Distance from patient's residence to the dental school	NS	NS	NS
60+ miles			
31–60 miles			
Less than or equal to 30 miles			
Types of providers			
Faculty member	3.75 (2.23–6.33)**	3.17 (2.09–4.81)**	3.21 (2.17–4.77)**
Resident	1.40 (0.79–2.50)	1.08 (0.69–1.70)	1.11 (0.71–1.73)
Pre‐doctoral student	Ref.	Ref.	Ref.
Calendar‐period effects	NS	NS	NS
Pre‐Covid baseline period (2008–2019)			
Covid‐19 disruption period (2020–2021)			
Post‐Covid recovery period (2022–2024)			
Number of daily medications	NS	NS	Not included
0–3			
4–10			
11+			
BMI level (kg/m^2^)	NS	Not included	Not included
<30 (Non‐obesity)			
≥30 (Obesity)			
Number of teeth present	NS	NS	Not included
Tobacco use	NS	NS	NS
Yes			
No			
Presence of diabetes	NS	NS	NS
No			
Yes			
Alcohol addiction	NS	NS	NS
Yes			
No			
Drug addiction	NS	NS	Not included
Yes			
No			
Presence of heart disease	NS	NS	NS
No			
Yes			
Presence of dry mouth			
Yes			
No	NS	Not included	Not included
−2 loglikelihood	888.7	1222.4	1230.7
AIC	930.7	1260.4	1254.7
Hosmer and Lemeshow Goodness‐of‐Fit Test (*p* value)	(0.692)	(0.420)	(0.569)

*Notes*: Results of logistic regression models evaluating factors associated with having ≥2 annual preventive dental. “Ref” indicates the reference category.

** *p* < 0.05 was considered statistically significant, and “NS” indicates non‐significant.

^a^ Full model adjusted for demographic and clinical variables, and calendar period effect.

^b^ Model adjusted for demographic and clinical variables, and calendar period effect, excluding two variables with 30%+ missing values.

^c^ Model adjusted for calendar period effect and variables (*p* < 0.05) in the simple logistic regression analysis.

**FIGURE 2:**
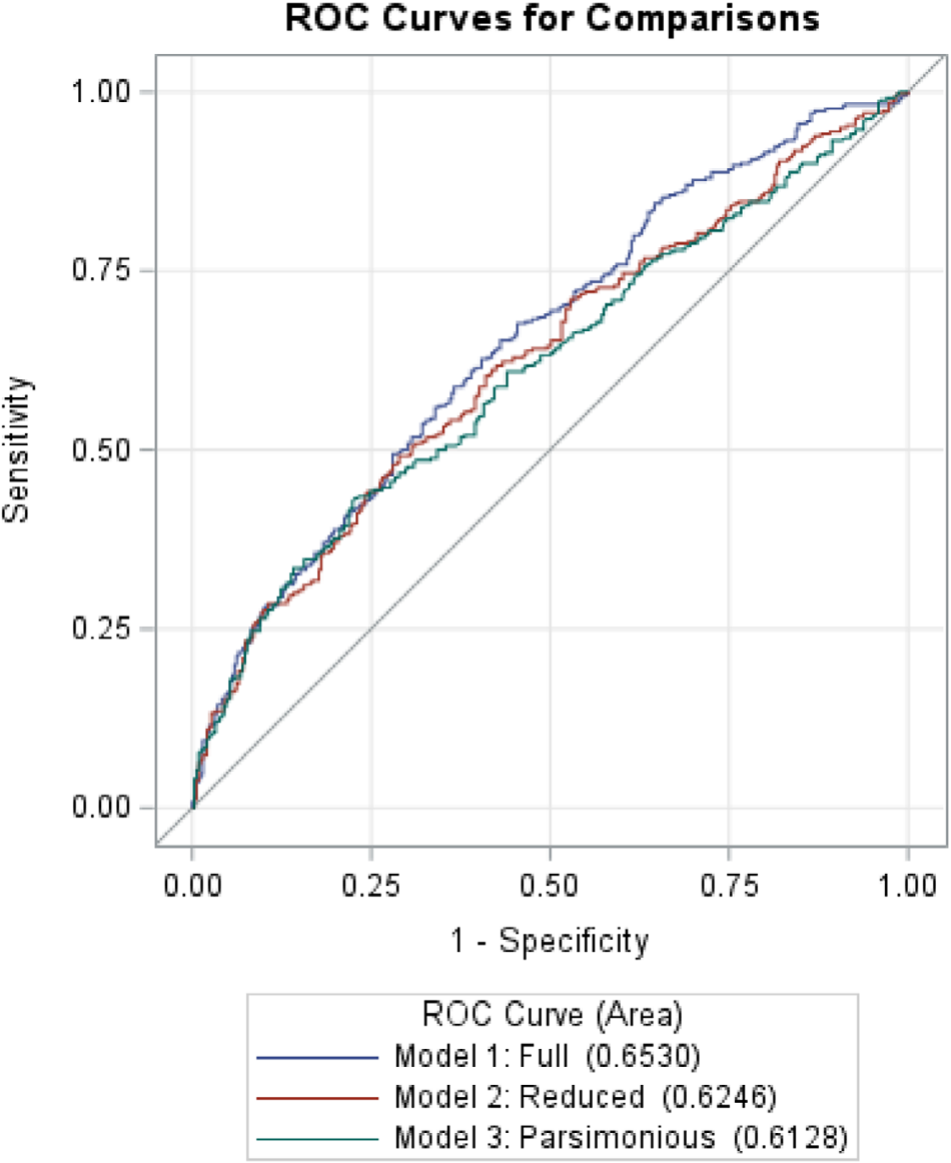
ROC curves and AUC comparisons among Models 1–3 (DeLong Test).

Discrimination was assessed using the area under the receiver operating characteristic (ROC) curve (AUC), as shown in Figure 2. Model 1 yielded the highest AUC (0.653; 95% CI: 0.612–0.694), indicating modest but superior discriminative ability compared to Model 2 (AUC 0.625; 95% CI: 0.583–0.666) and Model 3 (AUC 0.613; 95% CI: 0.571–0.655). Statistical comparisons using DeLong's test confirmed that Model 1 significantly outperformed Model 3 (*p* = 0.019), whereas differences between Models 1 and 2 (*p* = 0.071) and between Models 2 and 3 (*p* = 0.22) were not statistically significant. These results suggest that the full covariate set in Model 1 provides modest improvement in predictive discrimination over other two models.

Across all three models, provider type emerged as the strongest predictor. Patients treated by faculty providers had significantly higher odds of ≥2 preventive visits per year compared to those seen by pre‐doctoral students. In Model 1, the adjusted odds ratio (OR) was 3.75 (95% CI: 2.23–6.33; *p* < 0.001), and this effect remained consistent in Model 2 (OR = 3.17; 95% CI: 2.09‐4.81; *p* < 0.001) and Model 3 (OR = 3.21; 95% CI: 2.17–4.77; p < 0.001)). No statistically significant differences were found between resident and student providers. Age was a statistically significant predictor in Model 1 (OR = 1.05; 95% CI: 1.01–1.08; *p* = 0.004) but not in Models 2 or 3. Other variables, including gender, distance to care, insurance type, chronic disease indicators (diabetes, heart disease), tobacco or alcohol use, and calendar period, were not independently associated with frequent dental visits in any model.

In sum, although Model 1 demonstrated the best fit and discriminatory ability, Model 3 performed comparably while using fewer predictors and a larger sample. Therefore, Model 3 may offer a pragmatic balance between model simplicity and predictive value for identifying patients more likely to maintain regular preventive dental care. No significant interactions were detected; therefore, interaction terms were excluded from the final model to improve model parsimony.

## 4 Discussion

In this study, 33.6% of older adults with self‐reported mental health conditions had at least one preventive dental visit per year. This finding aligns with previous research indicating that individuals with mental health conditions are less likely to seek dental care compared to those without such conditions [20], often resulting in unmet oral healthcare needs [20, 21]. It is important to note that studies in this area would benefit from more standardized methodologies to reduce heterogeneity and enhance comparability across epidemiological investigations. Despite these methodological challenges, the collective evidence underscores the importance of identifying the factors that influence healthcare‐seeking behaviors and recognizing mental health difficulties as a significant risk factor for poor oral health outcomes [20]. From a clinical perspective, the reduced utilization of preventive dental services among individuals with mental health conditions highlights the urgent need for more integrated approaches to care [22]—particularly the coordination of mental, physical, and oral healthcare services.

Based on multivariable logistic regression analyses (Table 3), provider type emerged as the most influential predictor of preventive dental care utilization across all models. Patients treated by faculty members had significantly higher odds of receiving two or more preventive visits per year compared with those seen by predoctoral students (Model 1: OR = 3.75, 95% CI: 2.23–6.33; Model 3: OR = 3.21, 95% CI: 2.17–4.77; *p* < 0.001). No significant differences were found between resident and student providers. This suggests that faculty providers, who typically offer greater clinical experience and continuity of care, may play a crucial role in promoting preventive visit adherence among older adults with mental health conditions. The stability and sustained relationships fostered by faculty may enhance trust, patient education, and consistent follow‐up—factors known to improve care adherence and oral health outcomes in older adult populations.

Age was another significant factor in the fully adjusted model (Model 1: OR = 1.05; 95% CI: 1.01–1.08; *p* = 0.004), indicating that within this older population, increasing age was associated with a slightly higher likelihood of returning for preventive visits. This finding challenges the common assumption that advancing age leads to reduced care‐seeking behavior due to physical, psychological, or financial barriers. One possible explanation is that older adults managing multiple chronic conditions may have more frequent healthcare interactions overall, creating more opportunities for care coordination and reinforcement of preventive dental behaviors.

Although other variables—including distance to care, comorbidities (diabetes, heart disease), and behavioral factors (tobacco or alcohol use)—were associated with preventive‐visit frequency in unadjusted analyses (Tables 1 and 2), they were not independently significant in the multivariable models. This attenuation likely reflects confounding among predictors, where the effects of variables such as chronic conditions are explained by age and provider type once considered together. The calendar‐period effect, introduced to account for potential operational or behavioral shifts during the COVID‐19 pandemic (2008–2019, 2020–2021, 2022–2024), was also non‐significant across all models, suggesting that preventive‐care utilization among this population remained relatively stable over time.

Model performance indicators further support the robustness of these findings. Model 1 demonstrated the best overall fit (−2 log‐likelihood = 888.7; AIC = 930.7) and highest discriminative ability (AUC = 0.653, 95% CI: 0.612–0.694), whereas Models 2 and 3—with larger samples but fewer predictors—showed comparable calibration and only slightly lower discrimination (AUC = 0.625 and 0.613, respectively). The non‐significant Hosmer–Lemeshow tests (*p* = 0.42–0.69) confirmed good model fit. Although Model 1 provided modestly higher predictive accuracy, Model 3 offered a more parsimonious framework suitable for practical applications. No significant interactions were detected, and interaction terms were excluded to improve model simplicity and interpretability.

Together, these findings emphasize that both provider experience and age are key determinants of preventive dental visit frequency among older adults with mental health conditions. Dental schools and clinics serving older adults could improve care continuity and preventive‐care adherence by increasing faculty involvement in patient management and by training students to engage effectively with older patients with complex medical or psychological profiles. Furthermore, the absence of strong effects from other demographic or clinical variables suggests that interventions targeting provider continuity and patient engagement may yield the most significant improvements in preventive care utilization in this population.

While this study offers valuable insights into preventive dental care utilization among older adults with self‐reported mental health conditions, several limitations must be acknowledged. First, as a retrospective, single‐center study, the findings may not be generalizable to other settings or populations. In addition, selection bias is possible due to the exclusion of patients without preventive dental visits or without consistent annual visits, which may have resulted in an overrepresentation of individuals more engaged in preventive oral healthcare. Consequently, the observed associations may not fully reflect patterns among all older adults with mental health conditions.

Second, dichotomizing the outcome variable may have resulted in some loss of information, potentially obscuring finer differences in preventive dental visit frequency, but it allowed for a clinically meaningful comparison between patients meeting versus not meeting the recommended standard of care.

Third, our study relied on electronic health record (EHR) data, with mental health conditions identified through patient self‐report via the intake question: “Do you have, or have you had mental health issues?” Medication lists and problem lists were not used for classification. While self‐reported data may introduce some misclassification, this approach reflects the patient‐reported status recorded in the EHR, which is commonly used in clinical practice and research. Plausibility checks were conducted by examining consistency across repeated visits and reviewing available clinical notes where possible. Nevertheless, the use of EHR data introduces inherent limitations, including potential inaccuracies, incomplete documentation, and variation in data entry practices, which may lead to misclassification or missing information. These limitations could result in under‐ or over‐estimation of associations between mental health status and preventive dental care utilization.

Fourth, the retrospective design limits the ability to draw causal inferences, and unmeasured confounding variables may have influenced the observed associations. Additionally, the study sample was drawn from a single dental institution, which may restrict the generalizability of the findings to broader populations or different care settings. Future research should investigate the underlying reasons why a substantial portion of this population did not attend two or more annual preventive visits. Potential barriers—such as financial hardship, limited transportation, or the impact of mental health symptoms on motivation and self‐care—warrant further exploration. Incorporating patient‐reported outcomes and qualitative methods, such as interviews or focus groups, could provide a more nuanced understanding of the factors shaping dental care utilization in this vulnerable population. Such approaches would complement the current findings and support the development of more targeted, patient‐centered interventions.

## 5 Conclusion

In this sample of older adults with self‐reported mental health conditions attending a dental school, approximately one‐third had at least one annual preventive dental visit during the observation period. Additionally, receiving care from faculty members significantly increased the likelihood of having these annual preventive dental visits.

## Ethics Statement

The authors declare that the study conforms to recognized ethical standards and was approved by the University of Iowa Institutional Review Board as reported in the text.

## Conflicts of Interest

The authors declare no conflicts of interest.
